# Radiation-enhancement of MDA-MB-231 breast cancer cell invasion prevented by a cyclooxygenase-2 inhibitor

**DOI:** 10.1038/bjc.2011.260

**Published:** 2011-07-26

**Authors:** B Paquette, H Therriault, G Desmarais, R Wagner, R Royer, R Bujold

**Affiliations:** 1 Department of Nuclear Medicine and Radiobiology, Faculty of Medicine and Health Sciences, Université de Sherbrooke; 2 Center for Research in Radiotherapy, 3001, 12e Avenue Nord, Sherbrooke, Quebec, Canada J1H 5N4

**Keywords:** breast cancer, cyclooxygenase-2, radiation therapy, invasion

## Abstract

**Background:**

Recent evidences support that radiation can promote the invasion of cancer cells. As interactions between cancer cells and surrounding stromal cells can have an important role in tumour progression, we determined whether an irradiation to fibroblasts can enhance the invasiveness of breast cancer cells. The role of cyclooxygenase-2 (COX-2), an inflammatory enzyme frequently induced by radiotherapy, was investigated.

**Methods:**

Irradiated 3T3 fibroblasts were plated in the lower compartment of invasion chambers and used as chemoattractant for non-irradiated human breast cancer cell MDA-MB-231, which are oestrogen receptor negative (ER(−)) and the oestrogen receptor positive (ER(+)) MCF-7 cells. Stimulation of COX-2 expression in irradiated 3T3 cells was measured by a semi-quantitative qPCR and western blot. Capacity of the major product of COX-2, the prostaglandin E2 (PGE_2_), to stimulate the production of the matrix metalloproteinase-2 (MMP-2) and cancer cell invasion were assessed with a zymography gel and invasion chambers.

**Results:**

Irradiation (5 Gy) of 3T3 fibroblasts increased COX-2 expression and enhanced by 5.8-fold the invasiveness of non-irradiated MDA-MB-231 cells, while their migration was not modified. Addition of the COX-2 inhibitor NS-398 completely prevented radiation-enhancement of cancer cell invasion. Further supporting the potential role of COX-2, addition of PGE_2_ has increased cancer cell invasion and release of MMP-2 from the MDA-MB-231 cells. This effect of radiation was dependant on the expression of membrane type 1 (MT1)–MMP, which is required to activate the MMP-2, but was not associated with the ER status. Although irradiated fibroblasts stimulated the invasiveness of MDA-MB-231 ER(−) cells, no enhancement was measured with the ER(+) cell line MCF-7.

**Conclusions:**

Radiation-enhancement of breast cancer cell invasion induced by irradiated 3T3 fibroblasts is not dependant on the ER status, but rather the expression of MT1–MMP. This adverse effect of radiation can be prevented by a specific COX-2 inhibitor.

Malignant microfoci scattered throughout the breast tissue are present in 39–63% of breast cancer patients who are clinically and mammographically suspected of having unifocal breast cancer (Lagios *et al*, 1981; Holland *et al*, 1985). To eliminate residual cancer cells scattered in the breast, the radiation dose distribution used in radiotherapy is designed to irradiate the whole breast and frequently the axillaries and supraclavicular lymphatic nodes as well. Consequently, a large volume of healthy tissue is exposed to radiation. Moreover, a fraction of the cancer cells scatter in the breast could not be eliminated since the radiation treatment aims at optimising long-term results with minimal adverse effects to normal tissues, and not to eliminate all cancer cells.

Induction of an acute erythema and fibrosis by radiation are common adverse effects observed in patients under-going radiotherapy treatment (Nystrom *et al*, 2004; Lind, 2006). Some studies suggest that up to 95% of patients treated with postoperative external radiotherapy for breast cancer will experience some kind of skin reaction (De Conno *et al*, 1991). Early inflammatory response can occur during the radiotherapy treatment, while late side-effects appear months or even years after treatment and can even be irreversible (Nystrom *et al*, 2004).

Cyclooxygenase-2 (COX-2) is a key enzyme in the inflammatory response, which mainly produces the prostaglandin E2 (PGE_2_) (Liang *et al*, 2003). Overexpression of COX-2 and PGE_2_ were associated with unfavourable prognoses in breast cancer patients (Leppa *et al*, 2004; Schmitz *et al*, 2006). This prostaglandin can be involved in cancer cell invasion by stimulating the expression of matrix metalloproteinase 2 (MMP-2) (Yao *et al*, 2004; Larkins *et al*, 2006). This latter protease is required to cross the basement membrane, which acts as a barrier to cancer cell invasion (Rooprai and McCormick, 1997; Fillmore *et al*, 2001).

As radiation can stimulate the activity of COX-2 (Liang *et al*, 2003), the potential role of radiation to increase the invasiveness of cancer cell was investigated. An enhancement of cancer cell invasion after their irradiation has been reported for pancreatic cancer cells (Qian *et al*, 2002; Ohuchida *et al*, 2004), glioma cells (Wild-Bode *et al*, 2001; Park *et al*, 2006), melanoma cells (Rofstad *et al*, 2004; Kaliski *et al*, 2005), rectal carcinoma cells (Speake *et al*, 2005) and colon carcinoma cells (Wang *et al*, 2000). These studies were designed to evaluate the invasiveness of irradiated cancer cells, which would survival after radiation treatment.

The objective of this study was to determine the potential role of irradiated fibroblasts in the invasiveness of the human breast cancer cell MDA-MB-231 and MCF-7, which are oestrogen receptor negative (ER(−)) and oestrogen receptor positive (ER(+)), respectively. Role of the membrane type 1 (MT1)–MMP in radiation-enhancement of breast cancer cell invasion was also assessed. As our hypothesis is based on a participation of COX-2, the ability of a COX-2 inhibitor to prevent radiation-enhancement of breast cancer cells invasion was determined.

## Materials and methods

### Chemicals and reagents

Unless otherwise indicated, all reagents were purchased from Sigma-Aldrich (Oakville, Ontario, Canada).

### Mammary cell culture

The human breast cancer cell lines MDA-MB-231 and MCF-7 as well as the 3T3 fibroblasts were obtained from the American Type Culture Collection (Manassas, VA, USA). They were maintained in minimum essential medium (MEM) supplemented with sodium pyruvate (1  mM), 10% FBS, penicillin (50 units ml^–1^) and streptomycin (50 *μ*g ml^–1^).

### Cancer cell invasion and migration assay

For the invasion assay, the 3T3 fibroblasts (2.5 × 10^4^) plated in 24-well plates were incubated for 20 h in MEM medium supplemented with 10% FBS. The culture medium was then removed, cells rinsed twice with PBS and MEM medium supplemented with 0.1% BSA was added. Cells were irradiated using a ^60^Co source (Gammacell 220, Nordion, Canada) at a dose of 5 Gy. Non-irradiated cells were used as control. These different conditioned media were used as chemoattractant in the lower compartment of invasion chambers (Becton Dickinson Biosciences, Bedford, MA, USA). Non-irradiated MDA-MB-231 and MCF-7 cells harvested with cell dissociation solution were added (4 × 10^4^) to the upper compartment of the invasion chambers right after irradiation of the 3T3 cells in the lower compartment or 24 h later. For the invasion assays regarding the role of the MT1–MMP, the MT1–MMP antibody (Abcam, Cambridge, MA, USA) was added to a final concentration of 10 *μ*g ml^–1^ to the MDA-MB-231 cells 30 min before the invasion assay at 37 °C. To assess the role of COX-2, the invasion assay was repeated with 3T3 fibroblasts incubated with 10 *μ*M NS-398 (Cayman Chemical, Ann Arbor, MI, USA) 1 h before their irradiation. Breast cancer cells that had crossed the Matrigel and the porous membrane 24 h later were fixed, stained and counted under the microscope.

Regarding the migration assay, the same experimental conditions were used except that 1 × 10^4^ MDA-MB-231 cells were plated in the upper compartment of the migration chamber, which were not coated with a layer of Matrigel (Becton Dickinson Biosciences). Each experimental condition was performed in duplicate and repeated four times.

### MMP-2 analysis by zymography gel

The MDA-MB-231 cells (1.25 × 10^5^ in 12-well plate) were incubated on a layer of Matrigel (diluted 1/5) in MEM supplemented with 10% FBS for 18 h. The culture media was then removed, cells washed twice with PBS and PGE_2_ was added at the concentration indicated. Conditioned media containing the MMP-2 was analysed 24 h later by zymography gel as previously done in our laboratory (Paquette *et al*, 2007).

### Extraction of PGE_2_

3T3 fibroblasts (2.5 × 10^5^) plated in six-well plates were incubated for 24 h in MEM medium supplemented with 10% FBS. The culture medium was then removed, cells rinsed twice with PBS and MEM medium supplemented with 0.1% BSA was added. When indicated, 3T3 cells were incubated with 10 *μ*M NS-398 1 h before irradiation (5 Gy). The internal standard PGE_2_d_4_ (Cayman) at a concentration of 10 ng ml^–1^ was added to conditioned media of 3T3 cells isolated 24 h after the irradiation. Butylhydroxytoluene (10%) was added to prevent peroxidation by free radicals, and the samples were stored at −20 °C. Prostaglandins extraction was made according to Yang *et al* (2002). Briefly, 1 ml of acetone was added to cell supernatant, mixed and centrifuged at 1800 **g** for 10 min at 4 °C. Supernatant was then transferred to another tube with 1 ml hexane: ethyl acetate (1 : 1) and 30 *μ*l of 2 M formic acid. After being mixed and centrifuged, the upper layer was transferred to a conic tube for evaporation into a SpeedVac Concentrator (Sarant, Hicksville, NY, USA). Samples were reconstituted in 200 *μ*l methanol:10 mM ammonium acetate buffer, pH 8.5 (70 : 30) before liquid chromatography/tandem mass spectrometry (LC/MS/MS) analysis. All extraction procedures were performed under low-light and low-temperature conditions to minimise potential photo oxidation or thermal degradation of eicosanoid metabolites.

### PGE_2_ analysis by LC/MS/MS

PGE_2_ was quantified by LC/MS/MS using an API 3000 mass spectrometer (Applied Biosystem, Foster City, CA, USA) equipped with a Sciex turbo ion spray (AB Sciex, Toronto, Ontario, Canada) and Shimadzu pump and controller (Kyoto, Japan). Prostaglandins were chromatographically resolved using column Kromasil 100-3.5C18 150 × 2.1 mm (Eka Chemicals AB, Brewster, NY, USA). A linear acetonitrile gradient from 45% to 90% for 12 min at a flow rate of 200 *μ*l per min was used to achieve baseline resolution for compounds of interest. The mobile phase consisted of water buffered with 0.05% of acetic acid (A) and acetonitrile 90% with acetic acid 0.05% (B). Injection volume was 10 *μ*l per samples, which were kept at 4 °C during the analysis. Individual products were detected using negative ionisation and the monitoring of the transition *m/z* 351 → 271 for PGE_2_ and 355 → 275 for PGE_2_d_4_ at collision energy of −25 V.

### COX-2 mRNA quantified by a semi-quantitative PCR (qPCR)

3T3 fibroblasts (2.5 × 10^5^) plated in six-well plates were incubated for 24 h in MEM medium supplemented with 10% FBS. The culture medium was then removed, cells rinsed twice with PBS and MEM medium supplemented with 0.1% BSA was added. The 3T3 cells were irradiated (5 Gy) and the total RNA was extracted 24 h later. Total RNA extractions were performed on cell pellets with the Absolutely RNA Microprep Kit (Stratagene, La Jolla, CA, USA) as recommended by the manufacturer, except that DNAse treatments were done at 37 °C. RNA quality and presence of contaminating genomic DNA was verified as previously described (Brosseau *et al*, 2010). RNA integrity was assessed with an Agilent 2100 Bioanalyzer (Agilent Technologies, Mississauga, Ontario, Canada). Reverse transcription was performed on 2 *μ*g total RNA with Transcriptor reverse transcriptase, random hexamers, dNTPs (Roche Diagnostics, Laval, Quebec, Canada), and 10 units of RNAseOUT (Invitrogen, Burlington, Ontario, Canada) following the manufacturer's protocol in a total volume of 20 *μ*l. All forward and reverse primers were individually resuspended to 20–100 *μ*M stock solution in Tris-EDTA buffer and diluted as a primer pair to 1 *μ*M in RNase DNase-free water (IDT, Coralville, IA, USA). Semi-qPCR reactions were performed in 10 *μ*l in 96-well plates on a Realplex2 thermocycler (Eppendorf, Mississauga, Ontario, Canada) with 5 *μ*l of 2 × FastStart Universal SYBR Green Master mix (Roche Diagnostics), 10 ng (3 *μ*l) cDNA and 200 nM final (2 *μ*l) primer pair solutions. The following cycling conditions were used: 10 min at 95 °C; 50 cycles: 15 s at 95 °C, 30 s at 60 °C and 30 s at 72 °C. Relative expression levels were calculated using the qBASE framework (Hellemans *et al*, 2007) and the housekeeping genes *UBC, HPRT1* and *GAPDH* for mouse cDNA. Primer design and validation was evaluated as described elsewhere (Brosseau *et al*, 2010). In every qPCR run, a no template control was performed for each primer pair and these were consistently negative. Primer sequences: *COX-2*; sense primer 5′-TGGTTTTGTGCTGGCCTGGTA-3′, antisense primer 5′-TTCGAAGTTCAGCCTGGCAAGT-3′ *UBC*; sense primer 5′-CGTCGAGCCCAGTGTTACCACCAAGAAGG-3′, antisense primer 5′-CCCCCATCACACCCAAGAACAAGCACAAG-3′ *HPRT1*; sense primer 5′-GCTTGCTGGTGAAAAGGACCTCTCGAAG-3′, antisense primer 5′-CCCTGAAGTACTCATTATAGTCAAGGGCAT-3′ *GAPDH*, sense primer 5′-TGACGTGCCGCCTGGAGAAA-3′, antisense primer 5′-AGTGTAGCCCAAGATGCCCTTCAG-3′.

### Western blot analysis of COX-2

The 3T3 cells were irradiated (5 Gy) and the whole cell protein lysates were prepared 24 h later with lysis buffer containing 50  mM Tris-HCl, pH 7.5, 150  mM NaCl, 0.1% sodium dodecyl sulphate, 1% NP-40, 0.5% Na-deoxycholate and 5  mM EDTA supplemented with the protein inhibitor cocktail Complete Mini, EDTA-free (Roche, Indianapolis, IN, USA). Cellular debris was cleared by centrifugation and supernatants were aliquoted and stored at −80 °C for further use. Protein quantification assay was performed with a DC Protein Assay kit (Bio-Rad, Hercules, CA, USA). The protein extracts (50 *μ*g) were applied on a 12% polyacrylamide-SDS gel electrophoresed at 120 V during 3 h at 4 °C and transferred to a PVDF membrane (Millipore, Bedford, MA, USA) using the Mini Trans-Blot Cell (Bio-Rad) settled at 100 V for 1 h. The membrane was blocked with 8% reconstituted skim milk powder in TBST solution (10  mM Tris–HCl pH 7.5 containing 150 mM NaCl and 0.05% Tween 20). The blots were incubated with COX-2 antibody (Cayman) in blocking solution overnight at 4 °C. After washing with TBST, horseradish peroxidase-conjugated secondary antibodies (AbD Serotec, Raleigh, NC, USA) (1 : 10 000 dilution in TBST) were applied and the blots developed by the Enhanced Chemiluminescence Detection System (Perkin Elmer, Waltham, MA, USA). Levels of beta-actin immunocomplexes were used as an internal standard for equal loading.

### Statistical analysis

The data are expressed as the mean±s.d. Statistical analysis was performed using the Student's *t*-test, and a value of *P*<0.05 was considered significant.

## Results

### Irradiated 3T3 fibroblasts increases the invasion but not the migration of breast cancer cells

**Figure 1:**
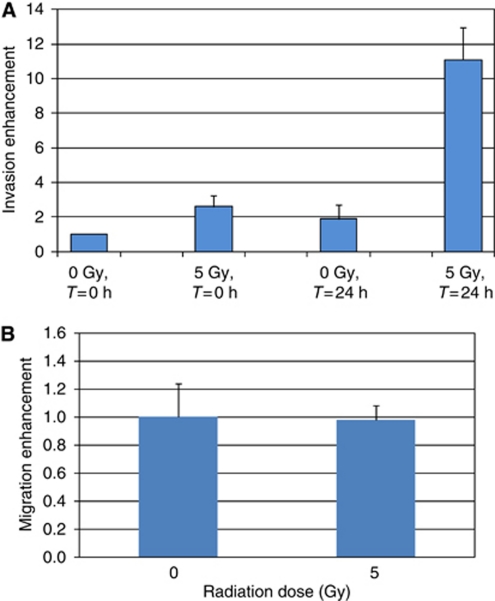
Irradiation of 3T3 fibroblasts increases the invasiveness of non-irradiated MDA-MB-231 cells. (**A**) 3T3 fibroblasts plated in the lower compartment of invasion chambers were irradiated at 5 Gy. Non-irradiated MDA-MB-231 cells were plated in the upper compartment of the invasion chambers either immediately after the irradiation, or 24 h later. The numbers of MDA-MB-231 cells, which have migrated through the layer of Matrigel were counted 24 h later. (**B**) 3T3 fibroblasts irradiated at 5 Gy were plated in the lower compartment of migration chambers, which were not coated with a layer of Matrigel. Non-irradiated MDA-MB-231 cells were plated in the upper compartment 24 h later. The numbers of MDA-MB-231 cells, which have crossed the porous filter were counted 24 h later. (**A**) 0 Gy *T*=0 h *vs* 5 Gy *T*=0 h, *P*=0.016; 0 Gy *T*=0 h *vs* 0 Gy *T*=24 h, *P*=0.06; 0 Gy *T*=24 h *vs* 5 Gy *T*=24 h, *P*=0.01. (**B**) 0 *vs* 5 Gy, *P*=0.91.

Irradiated 3T3 fibroblasts plated in the lower compartment of invasion chambers were used as chemoattractant to determine whether they can increase the invasiveness of non-irradiated breast cancer cells plated in the upper compartment of invasion chambers. When the non-irradiated MDA-MB-231 cells were plated right after irradiation of the 3T3 fibroblasts in the invasion chamber, their invasiveness increased by 2.6-fold±0.7 (Figure 1A, *T*=0 h, *P*=0.016). Irradiated 3T3 cells further stimulated cancer cell invasion when the invasion assay began 24 h after irradiation resulting in a larger number of MDA-MB-231 cells crossing the layer of Matrigel (Figure 1A, *T*=24 h, 5.8-fold±1.4, *P*=0.01). It is noteworthy that extending the incubation time to 24 h before to start the invasion assay did not significantly increase the invasiveness of MDA-MB-231 cells when the 3T3 cells were not irradiated (Figure 1A, 1.9-fold±0.8, *T*=24 h, *P*=0.06).

The effect of irradiated fibroblasts on the migration of the breast cancer cell MDA-MB-231 was assessed with migration chambers, which were similar to the invasion chamber but without the layer of Matrigel. The MDA-MB-231 cells were plated in the upper compartment 24 h after the irradiation. As shown on Figure 1B, the migration capacity of the MDA-MB-231 cells was not significantly affected by the irradiated fibroblasts compared with the non-irradiated control (ratio 5 Gy/0 Gy=0.98±0.19, *P*-value=0.91; Figure 1B).

### COX-2 inhibitor prevents radiation-enhancement of invasion

**Figure 2:**
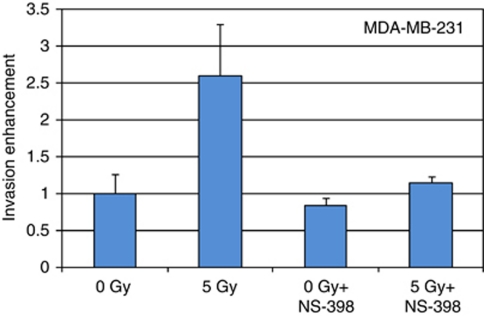
Prevention of radiation-enhancement of cancer cells invasion by a COX-2 inhibitor. The specific COX-2 inhibitor NS-398 (10 *μ*M) was added to the 3T3 cells 1 h before their irradiation (5 Gy). The invasion assays with the non-irradiated MDA-MB-231 cells were then repeated as previously described. 0 *vs* 5 Gy, *P*=0.01; 0 *vs* 0 Gy+NS-398, *P*=0.83; 0 *vs* 5 Gy+NS-398, *P*=0.51.

To determine the potential role of COX-2 in radiation-enhancement of cancer cells invasion, the specific COX-2 inhibitor NS-398 was added to the 3T3 fibroblasts 1 h before their irradiation. Invasion assays were then repeated with the MD-MB-231 cells (Figure 2). The COX-2 inhibitor completely prevented radiation-enhancement of cancer cells invasion (ratio 5 Gy+NS-398/0 Gy=1.15-fold±0.09, *P*=0.51). Supporting the role of PGE_2_, addition of the COX-2 inhibitor NS-398 to the 3T3 cells resulted in a drastic reduction of PGE_2_ production (Table 1). The effect of NS-398 on cancer cell invasion was specific to the irradiated fibroblasts because this inhibitor did not affect the invasion capacity of control MDA-MB-231 cells incubated with non-irradiated 3T3 fibroblasts (Figure 2).

### Effect of PGE_2_ on cancer cell invasion

**Figure 3:**
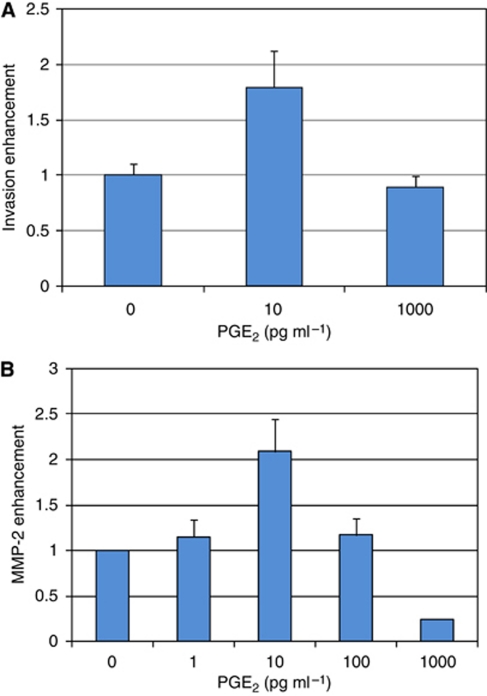
Modulation by PGE_2_ of invasion capacity of MDA-MB-231 cells and the release of MMP-2. (**A**) PGE_2_ was added to the MDA-MB-231 cells plated in the upper compartment of the invasion chambers. The invasion assay was then repeated as previously described. (**B**) PGE_2_ was added at the concentration indicated to MDA-MB-231 cells plated on a layer of Matrigel. The conditioned media containing the MMP-2 was analysed 24 h later by zymography gel. (**A**) 0 *vs* 10 pg ml^–1^, *P*=0.02; 0 *vs* 1000 pg ml^–1^, *P*=0.21. (**B**) 0 *vs* 10 pg ml^–1^, *P*=0.026.

The major product of COX-2 is the prostaglandin PGE_2_. This prostaglandin was added to the MDA-MB-231 cells plated in the invasion chamber to determine whether it can increase the invasion ability of these cancer cells (Figure 3A). A biphasic effect of PGE_2_ was observed. At a concentration of 10 pg ml^–1^, PGE_2_ stimulated the invasiveness of the breast cancer cells MDA-MB-231 by 1.8-fold±0.33 (*P*=0.02), while exposure to the higher concentration (1000 pg ml^–1^) did not significantly modify the invasiveness of the MDA-MB-231 cells (0.9-fold±0.1, *P*=0.21).

### Stimulation of MMP-2 by PGE_2_

The MDA-MB-231 cells plated on a layer of Matrigel, as occurred in the invasion chamber, were incubated with PGE_2_ for 24 h. The conditioned media was analysed for its content in MMP-2 by gel zymography (Figure 3B). A biphasic effect of PGE_2_ on the release of the protease MMP-2 by MDA-MB-231 cells was measured. The highest stimulation obtained at a concentration of 10 pg ml^–1^ resulted in a 2.1-fold±0.45 (*P*=0.026) increase in MMP-2 production. Conversely, higher concentration of PGE_2_ (1000 pg ml^–1^) has decreased the release of MMP-2 from the MDA-MB-231 cells.

### Effect of radiation on COX-2 mRNA expression and production of PGE_2_

The 3T3 fibroblasts were irradiated at 5 Gy and 24 h later the level of COX-2 mRNA was determined by a semi-qPCR, while the quantity of COX-2 protein was estimated with a western blot assay. As shown on Table 2, irradiation of the 3T3 cells enhanced the expression of COX-2 by 1.45-fold compared with the non-irradiated 3T3 cells (*P*-value=0.019). Regarding the COX-2 protein, a small but not significant increase was measured. Supporting that radiation did not significantly modify the COX-2 activity, no increase in PGE_2_ production from the irradiated 3T3 cells was measured by a LC/MS/MS analysis performed 24 h after radiation exposure (Table 1).

### Role of ER

**Figure 4:**
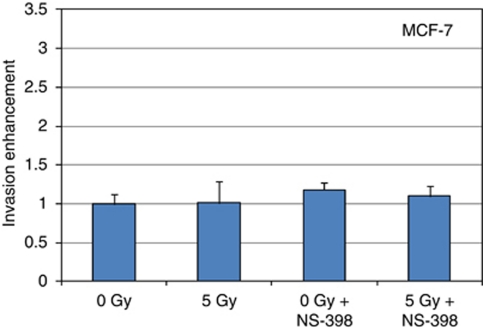
Effect of irradiated 3T3 fibroblasts on the invasiveness of MCF-7 cells. 3T3 fibroblasts plated in the lower compartment of invasion chambers were irradiated at 5 Gy. Non-irradiated MDA-MB-231 cells were plated in the upper compartment of the invasion chambers 24 h later. The numbers of MDA-MB-231 cells, which have migrated through the layer of Matrigel were counted 24 h later. 0 *vs* 5 Gy, *P*=0.01.

Ability of irradiated 3T3 fibroblasts to enhance the invasion of the MDA-MB-231 ER(−) was compared with breast cancer cell line MCF-7 ER(+) to assess the role of ER in radiation-enhancement of cancer cell invasion. The invasion assays demonstrated that while an important stimulation was measured with the MDA-MB-231 ER(−) cells (Figure 2), the invasiveness of the MCF-7 ER(+) was not increased by the irradiated 3T3 fibroblasts (Figure 4). An incubation with the COX-2 inhibitor NS-398 1 h before to start the invasion assay did modify the invasiveness of the MCF-7 incubated with irradiated and non-irradiated 3T3 fibroblasts (Figure 4).

### Association with MT1–MMP

**Figure 5:**
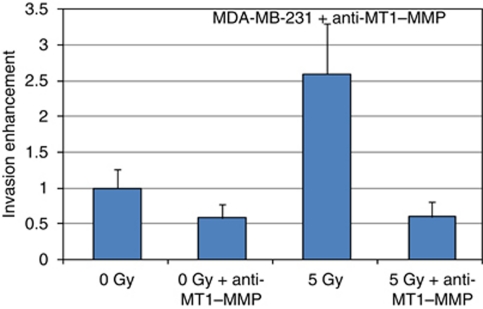
Reduction of the invasiveness of the MDA-MB-231 cells with the anti-MT1–MMP. Anti-MT1–MMP was added to the MDA-MB-231 cells plated in the upper compartment of the invasion chambers. The invasion assay was then repeated as previously described. 0 *vs* 0 Gy+anti-MT1–MMP, *P*=0.03; 5 *vs* 5 Gy+anti-MT1–MMP, *P*<0.01; 0 Gy+anti-MT1–MMP *vs* 5 Gy+anti-MT1–MMP, *P*=0.39.

In a previous study, we have quantified the expression of the MT1–MMP by a semi-quantification PCR in the breast cancer cell lines MDA-MB-231 and MCF-7 (Paquette *et al*, 2007). Although a high level MT1–MMP mRNA was found for the MDA-MB-231 cells, no significant quantity of this metalloproteinase was measured in the MCF-7 cells. To further assess the role of MT1–MMP in radiation-enhancement of cancer cell invasion, an anti-MT1–MMP was added to the MDA-MB-231 cells plated in the invasion chamber (Figure 5). A reduction of the invasiveness was observed when both the non-irradiated and the irradiated 3T3 fibroblasts were used as chemioattractant (0 *vs* 0 Gy+anti-MT1–MMP, *P*=0.03; 5 *vs* 5 Gy+anti-MT1–MMP, *P*<0.01). It is noteworthy that addition of the anti-MT1–MMP resulted to a similar level of invasion for the MDA-MB-231 cells incubated with either the irradiated or non-irradiated 3T3 fibroblasts (0 Gy+anti-MT1–MMP *vs* 5 Gy+anti-MT1–MMP, *P*=0.39).

## Discussion

Protocols for treating breast tumours by radiotherapy frequently included the whole breast and a large fraction of the chest to reach the axillaries and supraclavicular lymphatic nodes. Therefore, contrary to other cancers, a large number of normal cells received a significant radiation dose. As fibroblasts are normal component of the breast and their potential role in cancer progression has already been suggested (Camps *et al*, 1990; Ohuchida *et al*, 2004), their involvement in radiation-enhancement of breast cancer cell invasion was assessed.

Our study demonstrated that irradiation of fibroblasts increases the invasiveness of breast cancer cells MDA-MB-231 in an *in vitro* model, without affecting their ability to migrate. The clinical impact of this finding could be important because the interactions between cancer cells and surrounding stromal fibroblasts have been suggested to have a critical role in tumour invasion and metastasis (Camps *et al*, 1990; Ohuchida *et al*, 2004). Supporting the importance of fibroblasts, it was reported that a co-culture with non-irradiated fibroblasts significantly increased the invasiveness of pancreatic cancer cells, which was even further accelerated by a co-culture with irradiated fibroblasts (Ohuchida *et al*, 2004). More saliently, irradiated co-cultures of the low invasive colorectal cancer cell line LoVo with fibroblasts dramatically increased the invasiveness of these cancer cells (Speake *et al*, 2005).

To gain insights into the mechanisms involved in radiation-enhancement of cancer cell invasion, the potential role of COX-2 was assessed. This enzyme was chosen because it was associated with clinically more aggressive disease in addition to a decrease in survival of cancer patients (Gaffney *et al*, 2001; O’Connor *et al*, 2004). Making the link with cancer aggressiveness, Larkins *et al* (2006) have observed that COX-2 can stimulate breast cancer cell motility, invasion and MMP expression (Larkins *et al*, 2006). Furthermore, COX-2 is a key enzyme in the inflammatory response, which is induced by radiotherapy in almost all breast cancer patients. Therefore, as COX-2 expression correlates with tumour aggressiveness, adverse prognosis and that its expression can be stimulated by radiation, it follows that inhibition of COX-2 may have a therapeutic value.

In our study, inhibition of COX-2 by NS-398 completely prevented the enhancement of MDA-MB-231 cancer cell invasion induced by irradiated fibroblasts. As no PGE_2_ was measured after the addition of NS-398, this supports the activity of COX-2 was completely inhibited. It is interesting to note that invasiveness of the MDA-MB-231 cells incubated with the NS-398 came back to the same level measured in the assays done with non-irradiated fibroblasts. These results suggest that other factors independent of COX-2 activity also contributed to the invasion of these cancer cells, and they could work in association with irradiated fibroblasts to further enhance the invasion of breast cancer cell invasion.

In order to continue this study in an animal model, we have shown that a local irradiation before the implantation of breast cancer cells increased their invasiveness in surrounding normal tissue. This radiation-enhancement of cancer cell invasion was associated with an increase in COX-2 and MMP-2 expression (Lemay *et al*, 2011; Paquette *et al*, 2008). It remains to be determined whether a COX-2 inhibitor administrated in an animal model would prevent radiation-enhancement of cancer cell invasion.

COX-2 is a key enzyme induced during the inflammatory response, which produces the prostaglandin PGG_2_, a precursor of at least 5 prostaglandins and some thromboxanes. Among them, PGE_2_ was frequently associated to cancer progression. The 3T3 fibroblasts used in our study already secreted large among of PGE_2_ before their irradiation. Therefore, it might be not surprising that radiation could not further increase its production of PGE_2_. On the other hand, we showed that the breast cancer cell MDA-MB-231 released about 180 times less PGE_2_ than the 3T3 fibroblasts. We proposed that incubation of the MDA-MB-231 cells with the 3T3 fibroblasts in the invasion chamber produced a conditioned culture media where the final concentration of PGE_2_ results in an enhancement of cancer cell invasion.

The role of PGE_2_ in the invasion of MDA-MB-231 cells was associated to the concentration used during the invasion assay. Added at 10 pg ml^–1^ to the MDA-MB-231 breast cancer cells, PGE_2_ has increased cancer cell invasion and the release of MMP-2. However, a reduction of cancer cell invasiveness and MMP-2 released from the MDA-MB-231 cells were observed at a concentration of 1000 pg ml^–1^ PGE_2_. A similar biphasic effect of PGE_2_ was reported for the proliferation of fibroblasts and it was associated to the subtype of PGE_2_ receptors (EP1–4) activated (Timoshenko *et al*, 2003; White *et al*, 2008). Promitogenic effects of mid-range concentrations of PGE_2_ were mimicked by the EP3-selective agent (sulprostone) identifying EP3 as the key proliferative stimulatory receptor. Conversely, at higher concentrations of PGE_2_, inhibition of fibroblast proliferation was associated to the EP2 receptor (White *et al*, 2008). As the MDA-MB-231 breast cancer cells express all the four subtypes of PGE_2_ receptors, it remains to be determined whether the biphasic effect of PGE_2_ on their invasiveness and release of MMP-2 could be related to activation of different subtypes of its receptor.

We also assess the potential role of ERs in radiation-enhancement of cancer cell invasion. Oestrogen receptor has an important role in breast cancer progression. They are overexpressed in human breast cancers and associated with differentiated tumours and with a more favourable prognosis than ER(–) tumours, which are associated with an invasion behaviour and a shorter overall survival (Chen *et al*, 2002). Paradoxically, ER mediate the mitogenic action of oestrogens in human breast cancer cells, which suggests that locally produced oestrogen may have a role in stimulating neoplastic growth and development (Bulun *et al*, 1993). The promoter role of oestrogens in breast cancer has been evidenced by epidemiological studies indicating an increased incidence in women with prolonged exposure to oestrogens and a drastic decrease in incidence in women having nonfunctional ovaries (Pike *et al*, 1983).

Our study demonstrated that radiation-enhancement of breast cancer cell invasion was not associated with the ER. Although irradiated fibroblasts stimulated the invasiveness of MDA-MB-231 cells that are ER(−), no enhancement was measured with the ER(+) cell line MCF-7. Nevertheless, the potential role of oestradiol in the invasiveness of breast cancer cells should be further assessed because COX-2 can be associated with the production of oestradiol. Indeed, oestradiol is biosynthesised from androgens by the cytochrome P450 enzyme complex called aromatase, the product of the *CYP19* gene (Simpson *et al*, 1994). Although the levels of *CYP19* gene expression remained relatively constant in breast cancer tissue, increased level of CYP19 mRNA were found in advanced breast cancer tissue showing signs of invasion (Diaz-Cruz and Brueggemeier, 2006). A strong positive relationship was demonstrated between COX-2 and aromatase mRNA expression, and lends further support to the hypothesis that COX-2 is an upregulator of aromatase in breast tissue (Salhab *et al*, 2007). Thus, PGE_2_ produced via COX-2 may act locally in paracrine and autocrine manner to increase the biosynthesis of oestrogen by aromatase in hormone-dependent breast cancer development (Diaz-Cruz and Brueggemeier, 2006). Supporting the role of COX-2, studies have shown that COX inhibitors decrease aromatase activity in breast cancer cells and this effect starts at the transcriptional level. The precise role of COX-2 in the invasion of cancer cell ER(+) remains to be further investigated because COX-2 expression was also inversely associated with ER and it significantly correlated with worse survival (Zerkowski *et al*, 2007).

To further understand the role of oestrogens, we have previously studied their role in breast cancer cell invasion (Paquette *et al*, 2005). In this study, the ability of oestrogens to enhance the invasiveness of breast cancer cells was correlated to a subgroup of oestrogens, the hydroxyoestrogens. These oestrogens have been studied because the oestrogen metabolism is altered in malignant breast tumours that favour the accumulation of hydroxyoestradiol, which are known to generate free radicals (Abul-Hajj *et al*, 1988; Liehr, 2000; Paquette *et al*, 2001; Thibodeau *et al*, 2002). Using invasion chambers, oestradiol could not enhance the invasiveness of the MDA-MB-231 breast cancer cells. On the other hand, an important increase in breast cancer cell invasiveness was observed after adding the 4-hydroxyoestradiol. This increase was associated to the ability of 4-hydroxyoestradiol to generate free radicals, which can activate the MMP-2 and MMP-9 by removing their propeptide (Paquette *et al*, 2003). Therefore, it could be relevant to determine whether hydroxyoestrogens accumulated in breast tumours could increase the radiation-enhancement of cancer cell invasion in breast cancer cells ER(+) and ER(−).

Pro-MMP can also be activated by an enzymatic pathway. Indeed, conversion of inactive proMMP-2 into active MMP-2 can proceed through the formation of the complex proMMP-2/MT1–MMP/TIMP-2. This complex matures after binding to *α*_v_*β*_3_ integrin at the cancer cell surface, which concentrates the active MMP-2 at the migrating front (Brooks *et al*, 1996). Supporting the role of MT1–MMP, we demonstrated that the enhancement of MDA-MB-231 cell invasion induced by irradiated fibroblasts was prevented by incubating these cancer cells with a MT1–MMP antibody. A similar prevention of cancer cell invasion with a MT1–MMP antibody was previously reported by our team when MDA-MB-231 cells were incubated in invasion chambers containing irradiated Matrigel (artificial extracellular matrix) (Paquette *et al*, 2006). These two studies support that expression of MT1–MMP by breast cancer cells is required for the enhancement of their invasiveness induced by irradiation.

## Conclusion

Radiation-enhancement of breast cancer cell invasion induced by irradiated 3T3 fibroblasts is not dependant on the ER status, but rather on the expression of MT1–MMP. This adverse effect of radiation can be prevented by a COX-2 inhibitor. To better understand the involvement PGE_2_ in this process, the specific role of the four subtypes of PGE_2_ receptor should be assessed.

## Figures and Tables

**Table 1:** Production of PGE_2_ by MDA-MB-231 cells and irradiated 3T3 fibroblasts

	**PGE_2_ (pg ml^–1^)^a^**		
	**3T3 cells**		
**Dose (Gy)**	**Without NS-398**	**With NS-398**	**MDA-MB-231 cells**
0	1303 (±234)^b^	0.00^b^	7 (±3)
5	1151 (±127)	0.00	ND

Abbreviations: BSA=bovine serum albumin; LC/MS/MS=liquid chromatography/tandem mass spectrometry; ND=not determined; PGE_2_=prostaglandin E2.

a As assessed by LC/MS/MS. Cells irradiated in 0.1% BSA.

b Without NS-398: *n*=6, *P*-value=0.30. With NS-398: *n*=3, *P*-value=not applicable.

**Table 2:** Expression of COX-2 in irradiated 3T3 fibroblasts

**Enhancement ratio: 5 Gy/0 Gy**	
**COX-2 mRNA^a^**	**COX-2 protein^b^**
1.45 (±0.09)^c^	1.25 (±0.32)^c^

Abbreviations: COX-2=cyclooxygenase-2; qPCR=quantitative PCR.

a As assessed by a semi-qPCR.

b As assessed by western blot.

c *n*=3. COX-2 mRNA, *P*-value=0.019. COX-2 protein, *P*-value=0.14.
